# Dangers of Hypnotism

**Published:** 1890-06

**Authors:** 


### The Dangers of Hypnotism.

At Nuremburg a case of some public interest has recently been tried in the police court. A commercial traveler while in a restaurant told the waitress to look steadily at the white of his eye and hypnotized her. On the second occasion he repeated the experiment, but this time the sleep was so profound that a medical man had to be called, who had the utmost difficulty in rousing the girl. The commercial traveler was accordingly summoned to appear before the magistrates, and the severe sentence of eight day's imprisonment was passed on him, which will probably be efficient in checking similar performances in that region. In France the practice of hypnotizing people for amusement seems to be very common, and unpleasant consequences are reported. At a supper party in Paris recently one of the company hypnotized a girl and was unable to rouse her. She was consequently taken to the house of a medical man, and after a time she recovered consciousness. The whole party were taken in custody by the police, and were not released until next day. Even when hypnotism has been practiced by competent medical men for remedial purposes, unpleasant accidents and ulterior consequences have again and again occurred, so much so that recently an order has been issued by the French Government prohibiting surgeons in the army and navy from practicing it. It ought to be distinctly understood, both by the profession and the public, that hypnotism is not devoid of danger at the time, and not infrequently has permanently impaired the moral and emotional control of patients. A medical man is bound, before recommending hypnotism for a patient, to weigh the question as carefully as he would that of the advisability of administering an anæsthetic.—*Lancet.*

